# Ultrasound radiomics models improve preoperative diagnosis and reduce unnecessary biopsies in indeterminate thyroid nodules

**DOI:** 10.3389/fendo.2025.1615304

**Published:** 2025-07-10

**Authors:** Lu Chen, Yan Wang, Haoyu Jing, Rui Bao, Bin Sun, Mingbo Zhang, Yukun Luo

**Affiliations:** ^1^ Department of Ultrasound, The First Medical Center of Chinese People's Liberation Army (PLA) General Hospital, Beijing, China; ^2^ Graduate School Medical School of Chinese People's Liberation Army (PLA), Beijing, China

**Keywords:** indeterminate thyroid nodules, machine learning, radiomics model, ultrasound diagnosis, fine needle biopsy

## Abstract

**Purpose:**

Cytologically indeterminate thyroid nodules constitute 20–30% of fine-needle aspiration samples obtained from suspicious thyroid nodules. Over half of patients with indeterminate thyroid nodules undergo diagnostic surgery; however, 60–80% of excised nodules are benign. While some radiomics studies have built models to enhance the diagnostic efficacy of thyroid nodules, few have focused on indeterminate thyroid nodules with confirmed pathological results. We aimed to develop and evaluate ultrasound radiomics models to improve the diagnosis of indeterminate thyroid nodules and reduce unnecessary surgeries.

**Methods:**

We retrospectively analyzed ultrasound images of 197 indeterminate thyroid nodules with definitive pathological results. Regions of interest were manually delineated using 3-Dimensional Slicer software, and radiomics features were extracted using Pyradiomics software. Ultrasound radiomics feature selection and dimensionality reduction were performed using univariate analysis and the least absolute shrinkage and selection operator method. Independent training (n=136) and validation (n=61) cohorts were used to develop three radiomics models. Model performance was evaluated using receiver operating characteristic analysis and compared to two existing assisted diagnostic tools and two junior radiologists.

**Results:**

The Radunion model achieved the highest performance, with 90.5% sensitivity, 56.8% specificity, 75.0% positive predictive value, 80.7% negative predictive value, and 76.6% accuracy. The Radsize model minimized biopsies by 21.1%, reducing the rate from 48.9% to 13.8%. These models outperformed the ITS 100 system, Thynet deep learning-based tools (*p* < 0.05), and junior radiologists.

**Conclusion:**

Ultrasound radiomics models are promising, convenient, and accurate adjunct tools for predicting malignancy, improving junior radiologists’ diagnostic performance, reducing unnecessary biopsies, and enhancing diagnostic precision in clinical practice.

## 1 Introduction

Cytologically indeterminate thyroid nodules (ITNs) account for 20–30% of the fine-needle aspiration (FNA) samples from suspicious thyroid nodules (TNs) (1). These nodules correspond to Bethesda categories III–V, classified according to the Bethesda System for Reporting Thyroid Cytopathology (2). Bethesda III, IV, and V nodules carry a malignant risk of 13–30%, 23–34%, and 67–83%, respectively (2). Consequently, more than half of patients with ITNs opt for diagnostic surgery (3), although 60–80% of these excised nodules are benign on final pathological analysis (4, 5). Senior radiologists achieve excellent diagnostic efficacy for Bethesda V TNs using ultrasound (US) features (3). However, diagnosing Bethesda III and IV nodules remains challenging, despite reports that microcalcifications (6) and hypoechoic features (7) can predict malignancy. Grayscale US has significant limitations, exhibiting low diagnostic specificity (44–67.3%) (8–10) and high inter-observer variability (9, 11, 12), particularly for highly suspicious nodules (e.g., ACR TR4 and TR5). Differential diagnosis of ITNs requires a new solution to overcome the impact of radiologists, techniques, and equipment.

Radiomics has emerged as a promising approach for predicting the pathology, prognosis, and lymph node metastasis of TNs (13–18). Radiomics models based on US images demonstrate superior diagnostic efficacy compared with conventional US risk stratification systems (19). These models offer advantages, such as high accuracy (0.761–0.874) (14, 15, 20), lower intra-observer variability (14, 21), and reduced rates of unnecessary FNA procedures (3.1–37.7%) (19, 22) for TNs. However, previous models on common TN perform poorly for ITNs. The well-established artificial intelligence (AI) adjunct diagnostic tools have also demonstrated poor accuracy (e.g., 0.64 in accuracy for 88 Bethesda III nodules), despite achieving an AUC of 0.92 for common TNs (23). Few radiomics studies have focused on ITNs diagnosis or the diagnostic performance of ITN-specific radiomics models remains suboptimal, with area under curves (AUCs) ranging from 0.64 to 0.74 (23–25). A proportion of ITN patients undergo guideline-recommended follow-up observation or ablative minimally invasive treatment, making it difficult to collect ITNs with definitive cytopathology and postoperative histopathology. Due to the absence of such ITNs in training data, pilot studies propose that the efficiency of radiomics models could improve if trained specifically on ITN US images (24, 25). High indices, such as a negative predictive value (NPV) of 93.9% and a positive predictive value (PPV) of 93.8%, have been reported for Bethesda III nodules, indicating the potential utility of these models in supporting follow-up management of benign ITNs (26). However, such studies are limited, involving only dozens of ITNs. The critical questions remain unanswered regarding the diagnostic performance of ITN-specific radiomics models, their potential to enhance radiologists’ diagnostic accuracy, their role in reducing unnecessary aspiration biopsies, and their comparability to published AI adjunct diagnostic tools.

In this study, we aimed to address these gaps by developing an ITN-specific US radiomics model and comparing its performance with that of radiologists and published AI diagnostic tools. We assumed that radiomics could provide invisible and valuable features beyond radiologists’ observation. By combining conventional US and radiomics features of ITNs, the new method could improve the preoperative differentiation between benign and malignant ITNs. Using pathological diagnosis as the gold standard, we developed and evaluated the radiomics models in comparison with the Thynet online tool, the ITS 100 system, and two junior radiologists. Our aim was to improve the accuracy of preoperative ITN diagnosis and minimize unnecessary invasive interventions.

## 2 Methods

### 2.1 Patients

**Figure 1:**
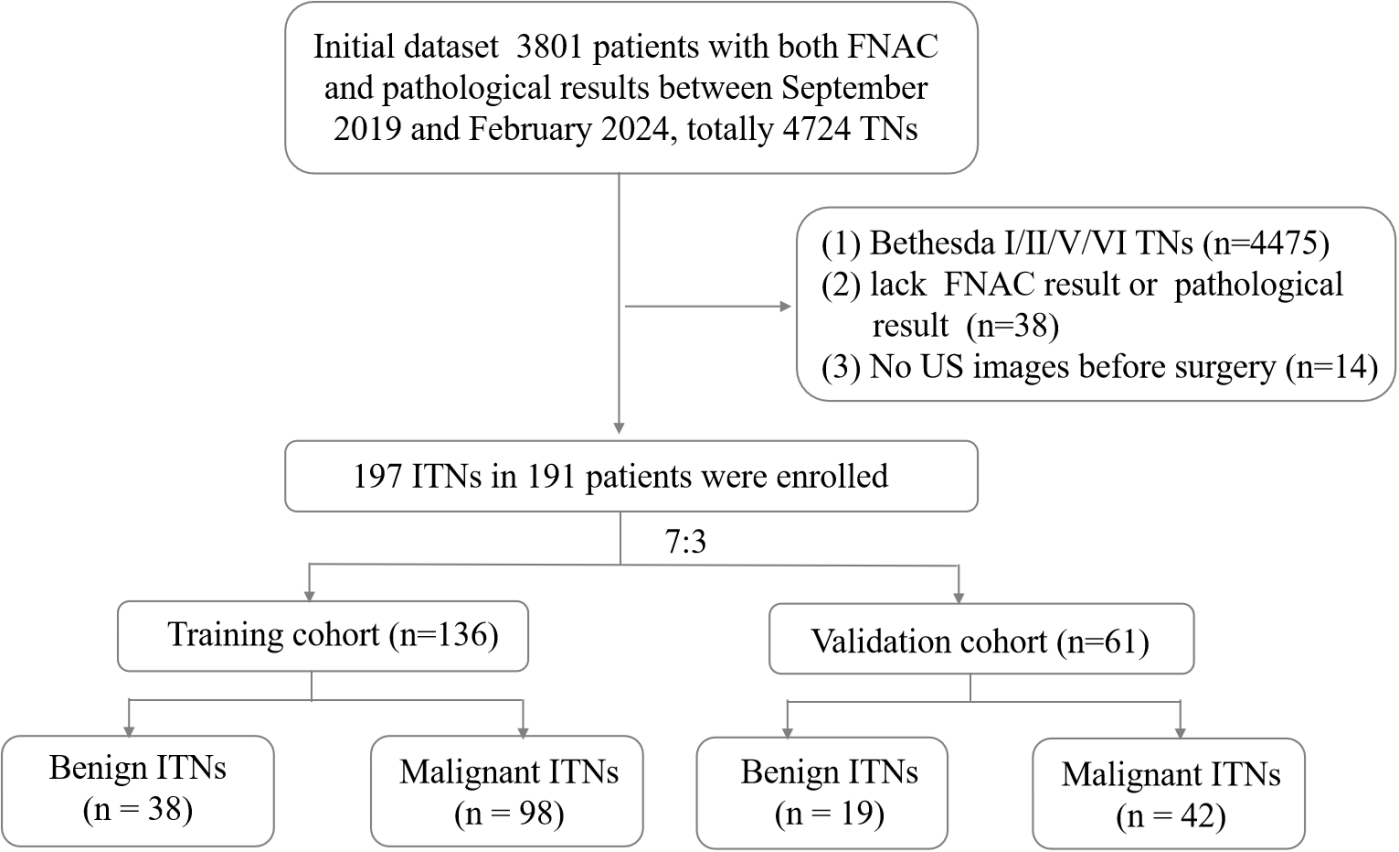
Flowchart of patient enrollment. FNAC, fine-needle aspiration cytology; ITN, indeterminate thyroid nodules; TN, thyroid nodules; US, ultrasound.

This retrospective study was approved by the Institutional Ethics Committee of the hospital. All procedures were performed in compliance with relevant laws and institutional guidelines. Given the retrospective nature of the study, the requirement for informed consent was waived. We clarified that all data were anonymized before processing and the study adhered to the Declaration of Helsinki. Between September 2019 and February 2024, 3,801 patients with ITN who underwent both fine-needle aspiration cytology (FNAC) and pathological examinations were initially assessed. The inclusion criteria were as follows (1): a definitive histopathological diagnosis of the target nodule following surgery, (2) a FNAC classification of Bethesda III or IV, and (3) availability of B-mode US performed within 2 weeks before resection. The exclusion criteria were as follows: (1) an FNAC classification of Bethesda I, II, V, or VI, (2) absence of postoperative pathological results, and (3) unclear or missing US images of the target nodule. A flowchart outlining the inclusion and exclusion process is presented in **Figure 1**.

A total of 191 patients with 197 ITNs were included (median age: 48 years; range: 24–76 years; sex: 36 men, 155 women). Four patients presented with two ITNs, and one presented with three ITNs. The ITNs were randomly divided into two cohorts in a 7:3 ratio: a training cohort with 136 nodules (25 men and 109 women) and a validation cohort with 61 nodules (11 men and 48 women).

### 2.2 Clinical and US information

Clinical data, including age, sex, FNAC results, US images, and pathological diagnoses, were collected from medical records. US images were acquired using 3–15 MHz linear probes from 10 different manufacturers (Philips, Toshiba, Siemens, Vinno, Hitachi, Aloka, GE Healthcare, Supersonic, Mindray, and Esaote). For quality control, low-quality images with severe artifacts or significant image resolution reductions were removed by two senior radiologists with over 5 years of thyroid US experience. These radiologists evaluated the images for five ACR TI-RADS lexicon features (composition, echogenicity, shape, margin, and echogenic foci) and determined the ACR rating for each nodule. One senior radiologist with > 10 years of experience and two junior radiologists with < 3 years of experience retrospectively assessed all images to classify nodules as “benign” or “malignant” for comparative diagnostic efficacy analysis. The pathology results were scrutinized and confirmed by a senior pathologist. All radiologists and pathologist were blinded.

### 2.3 Feature selection and model building

The clinical variables and all test results were analyzed via univariate and multivariate analysis. Variables with *p-*values < 0.05 in both analyses were retained. Regions of interest (ROIs) were manually delineated on US images in PNG format using 3D Slicer software (version 5.6.2, https://www.slicer.org, Earth, TX, USA) (**Supplementary Figure 1**). To assess reproducibility, a radiologist re-delineated all US images twice within a 2-week interval. An intraclass correlation coefficient (ICC) > 0.7 was considered indicative of satisfactory inter-observer agreement. Resampling and z-score normalization were applied to ensure consistency across repeated results, with a resampled resolution of 1×1 mm^2^ per pixel. Radiomics features were extracted using Pyradiomics software (http://pyradiomics.readthedocs.io/en/latest/index.html) with the default setting, yielding 851 original features. Radiomics feature selection and dimensionality reduction were first conducted by selecting features with an inter-observer ICC > 0.7. Subsequently, the optimal regularization parameter (λ) for the least absolute shrinkage and selection operator (LASSO) method was determined using the minimum criteria. Then, feature selection was performed through 10-fold cross-validation. Finally, the variance inflation factors (VIFs) for the features selected by LASSO were calculated to avoid severe linear dependence. After feature selection, a radiomics score (RAD-score) was generated through a linear combination of the selected features. Calibration was assessed for the radiomics models, and decision curve analysis was performed to evaluate their clinical utility by quantifying net benefits at different threshold probabilities in the entire cohort. The methodology for feature extraction and analysis followed previously established protocols, as outlined in the referenced literature (27).

### 2.4 Performance comparison with thyroid AI diagnosis tools

Two dynamic AI-based US auxiliary diagnostic systems were utilized for comparative analysis: UAI-X Laboratory’s Thynet tools (accessible online with author permission) (23) and Ian Thyroid Solution 100 (ITS100) (Med AI Technology Co. Ltd, Wuxi, China). Both systems employ convolutional neural network deep learning algorithms to provide dichotomous predictions (benign or malignant) for each nodule. These tools were trained using a large dataset of thyroid US images from the Chinese population. Thynet represents an academic research tool, whereas ITS100 is a commercial product integrated into an US instrument. The diagnostic performance of the ITN radiomics models was evaluated in comparison with these AI systems.

### 2.5 Statistical analyses

Statistical analyses were conducted using SPSS (version 22.0; IBM Corp., Armonk, NY, USA) and R software (version 4.3.2; Vienna, Austria). The Shapiro–Wilk test was employed to assess the normality of data distribution. Continuous variables were expressed as means ± SD and range values. Pathology diagnosis served as the gold standard for evaluating diagnostic performance. The sensitivity, specificity, PPV, NPV, accuracy, unnecessary biopsy rate, and AUC were calculated for radiomics models, radiologists, and thyroid AI diagnosis tools. The unnecessary biopsy rate was defined as the proportion of benign nodules among those classified as requiring biopsy. AUCs were statistically compared using the DeLong test, while proportions were compared using the chi-squared tests or Fisher’s exact test, as appropriate. Statistical significance was defined as *p* < 0.05.

## 3 Results

### 3.1 Patient characteristics

**Figure 2:**
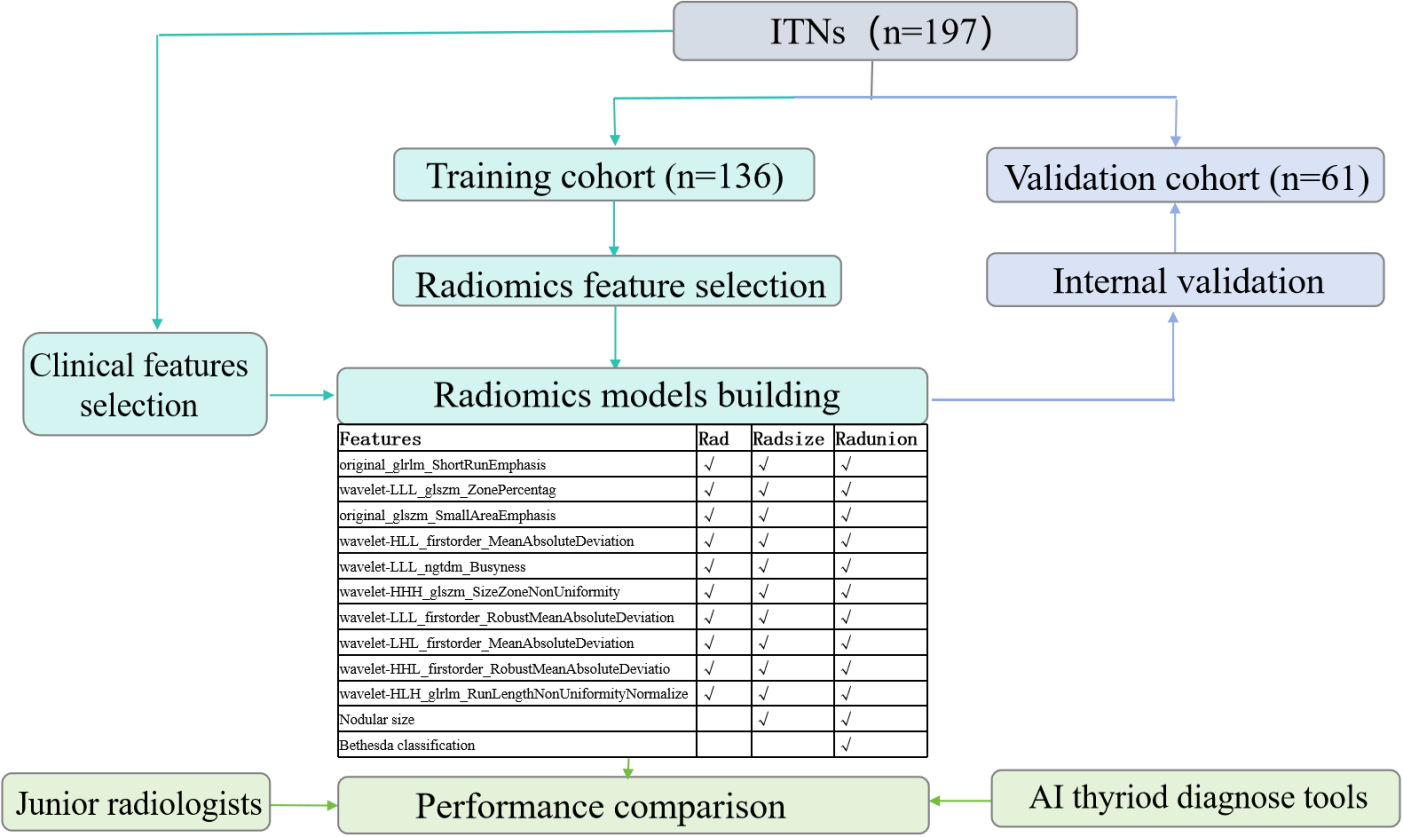
Radiomics diagnostic model study workflow.

This study evaluated 197 ITNs from 191 patients (36 men and 155 women), with a median age of 48 ± 11 (range: 24–76) years. The study flowchart is illustrated in **Figures 1**, **2**. **Tables 1**, **2** summarize the clinical and pathological characteristics of the training and validation cohorts. No significant differences were observed between these cohorts regarding pathological or US characteristics (all *p* > 0.05). The proportions of malignant nodules were 72.1% (98/136) and 68.9% (42/61) in the training and validation cohorts, respectively (*p* = 0.773). Malignant nodules exhibited significantly smaller diameters, higher nodular numbers, and elevated RadScores compared to benign nodules in both cohorts (all *p* < 0.05) (**Table 2**).

**Table 1:** Characteristics of ITNs in training and validation cohorts.

Features	level	Overall (n=197)	training cohort (n=136)	validation cohort (n=61)
Age,y (mean±SD)		48.15 ±11.21	48.32±11.30	47.77±11.10
Gender (%)	Female	161 (81.7)	111 (81.6)	50 (82.0)
	Male	36 (18.3)	25 (18.4)	11 (18.0)
Size,cm (mean±SD)		1.11±1.09	1.11±1.06	1.09±1.16
Bethesda (%)	BethesdaIII	136 (69.0)	91 (66.9)	45 (73.8)
	BethesdaIV	61 (31.0)	45 (33.1)	16 (26.2)
Invaded_capsule (%)	Negative	118 (59.9)	87 (64.0)	31 (50.8)
	Positive	79 (40.1)	49 (36.0)	30 (49.2)
TI-RADS (%)	2	5 (2.5)	4 (2.9)	1 (1.6)
	3	12 (6.1)	10 (7.4)	2 (3.3)
	4	74 (37.6)	53 (39.0)	21 (34.4)
	5	106 (53.8)	69 (50.7)	37 (60.7)
Composition (%)	Cystic and solid	8 (4.1)	7 (5.1)	1 (1.6)
	Solid	189 (95.9)	129 (94.9)	60 (98.4)
Echogenicity (%)	Hyperechoic/Isoechoic	22 (11.2)	15 (11.0)	7 (11.5)
	Hypoechoic	175 (88.8)	121 (89.0)	54 (88.5)
Border(%)	Clear	83 (42.1)	64 (47.1)	19 (31.1)
	Unclear	114 (57.9)	72 (52.9)	42 (68.9)
Margin (%)	Regular	76 (38.6)	55 (40.4)	21 (34.4)
	Irregular	121 (61.4)	81 (59.6)	40 (65.6)
Shape (%)	< 1	95 (48.2)	68 (50.0)	27 (44.3)
	> 1	102 (51.8)	68 (50.0)	34 (55.7)
Calcifications (%)	None	104 (52.8)	74 (54.4)	30 (49.2)
	Coarse/Peripheral calcification	14 (7.1)	9 (6.6)	5 (8.2)
	Punctate echogenic foci	79 (40.1)	53 (39.0)	26 (42.6)
CDFI (%)	No	123 (62.4)	80 (58.8)	43 (70.5)
	Yes	74 (37.6)	56 (41.2)	18 (29.5)
Number_of_nodules (%)	Single	9 (4.6)	8 (5.9)	1 (1.6)
	Multiple	188 (95.4)	128 (94.1)	60 (98.4)
BRAF_V600E (%)	Negative	149 (75.6)	103 (75.7)	46 (75.4)
	Positive	33 (16.8)	25 (18.4)	8 (13.1)
	Unknown	15 (7.6)	8 (5.9)	7 (11.5)
Metastasis (%)	No	143 (72.6)	99 (72.8)	44 (72.1)
	Yes	54 (27.4)	37 (27.2)	17 (27.9)
RAD-Score (mean±SD)		1.24 ± 1.88	1.24 ± 1.95	1.24 ± 1.73

Qualitative data were expressed as mean ± standard deviation or number and percentages (%), or median (25%–75% quantiles). ITNs, indeterminate thyroid nodules; TI-RADS, Thyroid Imaging Reporting and Data System.

**Table 2:** Characteristics of ITNs in the training and validation cohorts by pathology.

Features	level	Training cohort(n=136)				Validation cohort(n=61)		
		Benign (n=38)	Malignant (n=98)	*p*		Benign (n=19)	Malignant (n=42)	*p*
Age,y (mean±SD)		49.39±12.81	47.91±10.70	0.493		51.05±12.20	46.29±10.38	0.121
Gender (%)	Female	29 (76.3)	82 (83.7)	0.455		14 (73.7)	36 (85.7)	0.44
	Male	9 (23.7)	16 (16.3)			5 (26.3)	6 (14.3)	
Size,cm (mean±SD)		1.78±1.56	0.85±0.63	<0.001		2.04±1.71	0.67±0.31	<0.001
Bethesda (%)	BethesdaIII	13 (34.2)	78 (79.6)	<0.001	10 (52.6)	35 (83.3)	0.027	0.027
	BethesdaIV	25 (65.8)	20 (20.4)		9 (47.4)	7 (16.7)		
Invaded_capsule (%)	Negative	33 (86.8)	54 (55.1)	0.001	15 (78.9)	16 (38.1)	0.007	0.007
	Positive	5 (13.2)	44 (44.9)		4 (21.1)	26 (61.9)		
TI-RADS (%)	2	3 (7.9)	1 (1.0)	0.004	1 (5.3)	0 (0.0)	0.004	0.004
	3	5 (13.2)	5 (5.1)		2 (10.5)	0 (0.0)		
	4	19 (50.0)	34 (34.7)		10 (52.6)	11 (26.2)		
	5	11 (28.9)	58 (59.2)		6 (31.6)	31 (73.8)		
Composition (%)	Cystic and solid	2 (5.3)	5 (5.1)	1	0 (0.0)	1 (2.4)	1	1
	Solid	36 (94.7)	93 (94.9)		19 (100.0)	41 (97.6)		
Echogenicity (%)	Hyperechoic/Isoechoic	9 (23.7)	6 (6.1)	0.009	6 (31.6)	1 (2.4)	0.004	0.004
	Hypoechoic	29 (76.3)	92 (93.9)		13 (68.4)	41 (97.6)		
Border (%)	Clear	22 (57.9)	42 (42.9)	0.166	8 (42.1)	11 (26.2)	0.345	0.036
	Unclear	16 (42.1)	56 (57.1)		11 (57.9)	31 (73.8)		
Margin (%)	Regular	23 (60.5)	32 (32.7)	0.005	7 (36.8)	14 (33.3)	1	
	Irregular	15 (39.5)	66 (67.3)		12 (63.2)	28 (66.7)		
Shape (%)	< 1	27 (71.1)	41 (41.8)	0.004	12 (63.2)	15 (35.7)	0.085	0.24
	> 1	11 (28.9)	57 (58.2)		7 (36.8)	27 (64.3)		
Calcifications (%)	None	24 (63.2)	50 (51.0)	<0.001	9 (47.4)	21 (50.0)	0.041	
	Coarse/Peripheral calcification	7 (18.4)	2 (2.0)		4 (21.1)	1 (2.4)		
	Punctate echogenic foci	7 (18.4)	46 (46.9)		6 (31.6)	20 (47.6)		0.054
CDFI (%)	No	20 (52.6)	60 (61.2)	0.472	13 (68.4)	30 (71.4)	1	
	Yes	18 (47.4)	38 (38.8)		6 (31.6)	12 (28.6)		
Number_of_nodules (%)	Single	6 (15.8)	2 (2.0)	0.008	1 (5.3)	0 (0.0)	0.681	0.041
	Multiple	32 (84.2)	96 (98.0)		18 (94.7)	42 (100.0)		
BRAF_V600E (%)	Negative	33 (86.8)	70 (71.4)	0.132	17 (89.5)	29 (69.0)	0.113	
	Positive	3 (7.9)	22 (22.4)		0 (0.0)	8 (19.0)		1
	Unknown	2 (5.3)	6 (6.1)		2 (10.5)	5 (11.9)		
Metastasis (%)	No	32 (84.2)	67 (68.4)	0.099	17 (89.5)	27 (64.3)	0.085	0.681
	Yes	6 (15.8)	31 (31.6)		2 (10.5)	15 (35.7)		
RAD-Score (mean±SD)		-0.55±2.18	1.93±1.31	<0.001		0.42±2.11	1.61±1.40	0.012

Qualitative data were expressed as mean ± standard deviation or number and percentages (%), or median (25%–75% quantiles). ITNs, indeterminate thyroid nodules; TI-RADS, Thyroid Imaging Reporting and Data System.

### 3.2 Feature selection and RAD-Score development

Univariate analysis and multivariate analysis revealed that nodular size (*p* < 0.014), Bethesda classification (*p* < 0.038), and capsular invasion (*p* < 0.001) were significant variables with *p* < 0.05. Followed by an ICC > 0.7, there were 37 radiomics features selected using the LASSO method with the regularization parameter (λ) values of 0.034 (**Supplementary Figure 2a, b**). Finally, 10 features were included in the RAD-Score formula as VIF < 10 to avoid severe linear dependence (**Supplementary Figure 2c**). Among them, original_glrlm_ShortRunEmphasis showed negative relation with malignancy while wavelet-HLH_glrlm_RunLengthNonUniformityNormalized showed positive relation with malignancy, which both might be corresponding to unclear border and irregular margin in the US features.

Since capsular invasion is a postoperative variable and not suitable for preoperative diagnostic purposes, it was excluded from the radiomics models.

**Figure 3:**
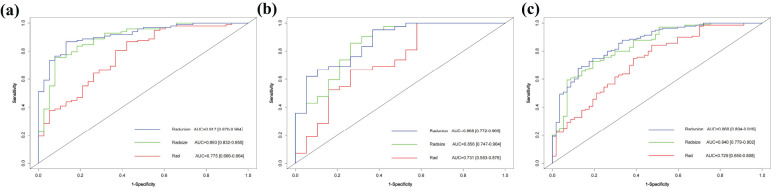
Receiver operating characteristic (ROC) curves of radiomic models in **(a)** training cohort, **(b)** validation cohort, and **(c)** entire cohorts.

**Figure 4:**
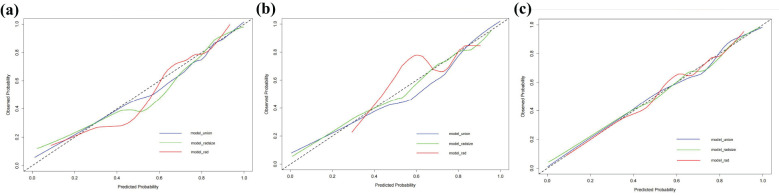
Calibration curves of radiomic models in **(a)** training cohort, **(b)** validation cohort, and **(c)** entire cohorts.

**Figure 5:**
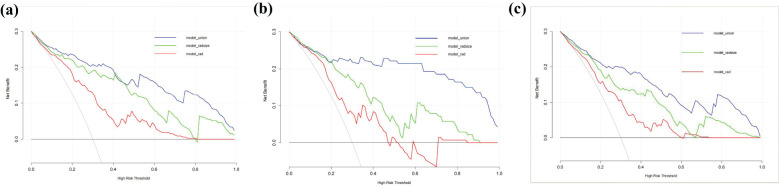
Decision curve analysis (DCA) of the radiomics models in predicting malignancy in thyroid nodules: **(a)** training cohort, **(b)** validation cohort, and **(c)** entire cohorts. The vertical axis measures standardized net benefit. The horizontal axis shows the corresponding risk threshold. The DCA results indicate that the Radunion model had a higher overall net benefit compared to the other models.

The RAD-Score for malignant nodules was significantly higher than that for benign nodules in the training ([1.93 ± 1.31] *vs*. [−0.55 ± 2.18], *p* < 0.001) and validation cohorts ([1.61 ± 1.40] *vs*. [0.42 ± 2.11], *p* = 0.012) (**Table 2**). The Rad model yielded AUCs of 0.775 (95% confidence interval [CI]: 0.686–0.864) in the training cohort (**Figure 3a**) and 0.731 (95% CI: 0.583–0.878) in the validation cohort (**Figure 3b**). Adding nodular size improved the model’s AUC to 0.893 (95% CI: 0.832–0.955) in the training cohort (**Figure 3a**) and 0.856 (95% CI: 0.747–0.964) in the validation cohort (**Figure 3**). Further addition of Bethesda classification resulted in the Radunion model with an AUC of 0.860 (95% CI: 0.804-0.916) for the entire cohort (**Figure 3c**). The Radsize and Radunion models significantly outperformed the Rad model (*p* < 0.001), although differences between the Radsize and Radunion models were not statistically significant (*p* > 0.001). The calibration curves of three radiomics models are shown in **Figure 4**, and the Radunion model showed the best calibration. Decision curve analysis indicated that the radiomics models were clinically useful, with the Radunion providing the greatest net benefit (**Figure 5**).

### 3.3 Diagnostic performance of models

**Figure 6:**
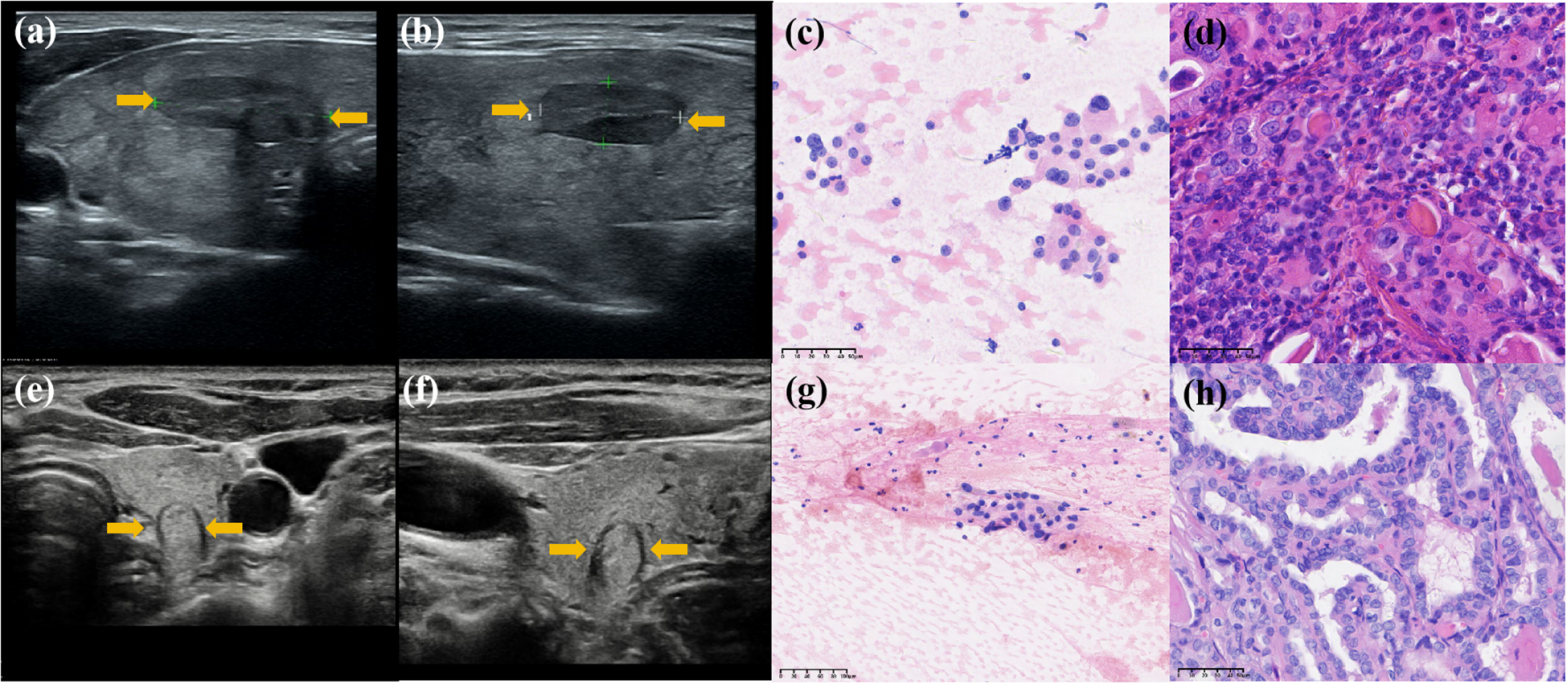
Diagnosis of two nodules: Case 1 was a hypoechoic nodule in the right lobe of the thyroid. **(a, b)** Transverse and longitudinal US images. **(c)** Bethesda IV after FNA, and **(d)** histopathological result: benign. Two AI models classified it as a “benign” nodule, while two junior radiologists assessed it as “malignant,” and all three radiomics models classified it as “benign.” Case 2 was a hypoechoic nodule in the left lobe of the thyroid. **(e, f)** Transverse and longitudinal US images. **(g)** Bethesda III after FNA, and **(h)** histopathological result: benign. Two AI models classified it as “malignant,” two junior radiologists assessed it as “benign,” and all three radiomics models classified it as “benign.” Its histopathological diagnosis is “benign.”.

Radiomics models demonstrated robust performance in distinguishing malignant TNs from benign ones, with the Radunion model achieving the highest accuracy of 85.3% in the training cohort (**Table 3**). The Radsize model had sensitivity, specificity, PPV, NPV, and accuracy rates of 71.4% (63.9−78.9%), 80.7% (70.5−90.9%), 90.1% (84.5−95.6%), 53.5% (42.9−64.0%), and 74.1% (68.0−80.2%), respectively. This model also reduced the unnecessary biopsy rate to 21.1% (8.1−34.0%) (**Table 4**). The Radunion model demonstrated the best overall performance, with sensitivity, specificity, PPV, NPV, and accuracy rates of 90.5% (85.2−95.8%), 56.8 (46.0−67.6%), 75.0% (67.8−82.2%), 80.7% (70.5−90.9%), and 76.6% (70.7−82.6%), respectively (**Table 4**). It also reduced overtreatment by 13−20% of false-positive cases. The accuracy of the ITS 100 system, Thynet online tools and two junior radiologists were 68.0%, 65.0%, 61.9% and 69.5%, respectively. Radiomics models outperformed the ITS 100 system and Thynet deep learning tools (*p* < 0.05), as well as two junior radiologists in terms of diagnostic accuracy (radiomics models *vs*. Junior radiologist 2, *p* < 0.05; radiomics models *vs*. Junior radiologist 1, *p* > 0.05) (**Table 4**). Two cases in **Figure 6** demonstrate that the radiomics models provided accurate and stable diagnoses among AI-based tools, and junior radiologists for two cases.

**Table 3:** The performance of the Rad, Radsize, Radunion models.

	Training cohort(n = 136)			Validation cohort(n = 61)			Entire data(n = 197)		
	rad	radsize	union	rad	radsize	union	rad	radsize	union
**sensitivity**	86.7% (80.0%-93.5%)	75.5% (67.0%-84.0%)	84.7% (77.6%-91.8%)	100%(100%-100%)	85.7%(75.1%-96.3%)	95.2%(88.8%-100.0%)	82.9% (76.6%-89.1%)	71.4% (63.9%-78.9%)	90.5% (85.2%-95.8%)
**specificity**	57.9% (42.2%-73.6%)	92.1% (83.5%-100%)	86.8%(76.1%-97.6%)	42.1%(19.9%-64.3%)	73.7%(53.9%-93.5%)	63.2%(41.5%-84.8%)	52.6% (39.7%-65.6%)	80.7% (70.5%-90.9%)	56.8% (46.0%-67.6%)
**PPV**	84.2% (77.0%-91.3%)	96.1% (91.8%-100%)	94.3%(89.5%-99.2%)	79.2%(68.3%-90.2%)	87.8%(77.8%-97.8%)	85.1%(74.9%-95.3%)	81.1% (74.7%-87.5%)	90.1% (84.5%-95.6%)	75.0% (67.8%-82.2%)
**NPV**	62.9% (46.8%-78.9%)	59.3%(46.8%-71.9%)	68.8%(55.6%-81.9%)	100%(100%-100%)	70.0%(49.9%-90.1%)	85.7% (67.4%-100.0%)	55.6% (42.3%-68.8%)	53.5% (42.9%-64.0%)	80.7% (70.5%-90.9%)
**f1_score**	85.4%	84.6%	89.2%	88.4%	86.7%	89.9%	82.0%	79.7%	82.0%
**accuracy**	78.7%(71.8%-85.6%)	80.1%(73.4%-86.9%)	85.3%(79.3%-91.2%)	82.0%(72.3%-91.6%)	82.0%(72.3%-91.6%)	85.2%(76.3%-94.1%)	74.1% (68.0%-80.2%)	74.1% (68.0%-80.2%)	76.6% (70.7%-82.6%)
**p_value**	<0.001	<0.001	<0.001	<0.001	<0.001	<0.001	<0.001	<0.001	<0.001
**auc**	0.775 (0.686-0.864)	0.893 (0.832-0.955)	0.917 (0.870-0.964)	0.731 (0.583-0.878)	0.856 (0.747-0.964)	0.868 (0.772-0.965)	0.729(0.650-0.808)	0.840(0.779-0.902)	0.860(0.804-0.916)
**Youden_index**	44.6%	67.6%	73.5%	42.1%	59.4%	58.4%	36.9%	53.5%	55.7%
**UFR**	55.2% (37.1%-73.3%)	10.3% (0.0-21.4%)	25.0% (6.0%-44.0%)	100% (100%-100%)	35.7% (10.6%-60.8%)	77.8% (50.6%-100.0%)	52.9% (39.2%-66.6%)	21.6%(10.3%-32.9%)	–

Rad model integrates only radiomics features, Radsize model integrates radiomics features and nodular size, Radunion model integrates radiomics features, nodular size and Bethesda classification. PPV, positive predictive value; NPV, negative predictive value; UFR, unnecessary biopsy rate. n=136/61/197 means the number of nodules in training cohort , validation cohort and entire cohort, respectively.

**Table 4:** Performance summary among AI tools, radiologists and two radiomic models.

model	Senior radiologist	Junior radiologist 1	Junior radiologist 2	ITS 100	Thynet	Radsize model	Radunion model
sensitivity	92.1% (87.7%-96.6%)	66.4%(58.6%-74.3%)	87.1%(81.6%-92.7%)	70.7%(63.2%-78.3%)	74.3%(67.0%-81.5%)	71.4% (63.9%-78.9%)	90.5% (85.2%-95.8%)
specificity	50.9% 37.9%-63.9%)	50.9%(37.9%-63.9%)	26.3%(14.9%-37.7%)	61.4%(48.8%-74.0%)	42.1%(29.3%-54.9%)	80.7% (70.5%-90.9%)	56.8 (46.0%-67.6%)
PPV	82.2% (76.2%-88.2%)	76.9%(69.3%-84.4%)	74.4%(67.7%-81.1%)	81.8%(74.9%-88.7%)	75.9%(68.8%-83.1%)	90.1% (84.5%-95.6%)	75.0% (67.8%-82.2%)
NPV	72.5% (58.7%-86.3%)	38.2%(27.2%-49.1%)	45.5% (28.5%-62.4%)	46.1%(34.8%-57.3%)	40.0%(27.6%-52.4%)	53.5% (42.9%-64.0%)	80.7%(70.5%-90.9%)
f1_score	86.90%	71.3%	80.3%	75.9%	75.1%	79.7%	82.00%
accuracy	80.2% (74.6%-85.8%)	61.9%(55.1%-68.7%)	69.5% (63.1%-76.0%)	68.0%(61.5%-74.5%)	65.0%(58.3%-71.6%)	74.1% (68.0%-80.2%)	76.6% (70.7%-82.6%)
p_value	<0.001	0.036	0.037	<0.001	0.036	<0.001	<0.001
UFR	71.8% (57.7%-85.9%)	37.3%(26.4%-48.3%)	70.0%(58.4%-81.6%)	34.9%(23.1%-46.7%)	47.8%(36.0%-59.6%)	21.1%(8.1%-34.0%)	_

ITS100, Ian Thyroid Solution 100, Thynet, online Thyroid AI auxiliary tools, Radsize model integrates radiomics features and nodular size, Radunion model integrates radiomics features, nodular size and Bethesda classification. PPV, positive predictive value; NPV, negative predictive value; UFR, unnecessary biopsy rate.

## 4 Discussion

In this study, we developed three radiomics models using US images of ITNs. The models were constructed as follows: the Rad model, based solely on radiomics features; the Radsize model, which incorporated nodular size and radiomics features; and the Radunion model, which included the Bethesda classification along with the Radsize model features. The models achieved diagnostic accuracies ranging from 74.1% to 85.3% across all ITN cohorts, outperforming both junior radiologists and two AI-assisted diagnostic tools. This demonstrates the potential of radiomics models to differentiate malignant from benign ITNs. Notably, the Radsize model reduced unnecessary biopsy rates by at least 13.8%, while the Radunion model could potentially spare 13%–20% of ITNs from diagnostic surgery prior to intervention.

Beyond radiomics features, our findings identified nodular size, Bethesda classification, and microscopic capsular invasion as significant predictive factors for ITN malignancy. Interestingly, none of the five ACR TI-RADS-recommended features (composition, echogenicity, shape, border, and echogenic foci) significantly predicted malignancy in ITNs within our cohort (28). This suggests a potential need to refine conventional diagnostic criteria for ITNs.

In our cohort, benign and malignant ITNs exhibited significant differences in nodule size, which corroborates findings by Xavier et al., who identified nodular size as a key factor in model development (25). The ACR guidelines associate larger nodules with higher malignancy risks, recommending FNA for nodules >2.5 cm in TR3 categories or follow-up for nodules <1.5 cm. However, our results showed that most malignant nodules were smaller, likely reflecting the increased prevalence of papillary thyroid microcarcinomas (<10 mm). Bethesda classification showed that Bethesda IV nodules were at higher risk of malignancy than Bethesda III nodules, which aligns with existing guidelines (2).

The Radsize model demonstrated significantly improved performance in both the training and entire cohorts compared to the Rad model. Furthermore, including the Bethesda classification in the Radunion model enhanced diagnostic precision, reducing the need for diagnostic surgery. Similarly, Grégoire et al. incorporated Bethesda classifications into logistic regression models for Bethesda III–V nodules, demonstrating comparable improvements (20). Although microscopic capsular invasion showed no preoperative diagnostic value, gross extrathyroidal extension diagnosed via preoperative US remains a key determinant for surgical planning in thyroid cancers (29).

The diagnostic accuracy of the Rad model was comparable to that of an SVM-based model by Chen et al. (74.1% *vs*. 71.8%) (26), which utilized clinical and sonographic features such as composition, echogenicity, margins, shape, echogenic foci, and nodule size in 194 ITNs (Bethesda III/IV/V). The AUC of the Radsize model outperformed that of the ResNet-50 model, which integrated radiomics features from 88 ITNs (0.840 *vs*. 0.740) (25), and was comparable to the multiple-modality models by Grégoire et al. (20), which combined clinical data with the Bethesda and French TI-RADS categories. These findings suggest that US radiomics may play an important role in enhancing the differential diagnosis of ITNs.

The Radunion model achieved an AUC of 0.860 and the highest accuracy of 76.6% among junior radiologists, the Thynet online tools, and the ITS 100 system. In contrast, the previous Thynet tool, based on a deep learning algorithm and trained on 22,354 US images, achieved an AUC of 0.922 (23) but yielded an accuracy of only 65% in our ITN cohort. Part of Thynet’s training set included Bethesda II or VI nodules, which lack the characteristic features commonly observed in ITN US images. Most training images were from surgical nodules with a high malignant potential, which may explain why the Thynet model was less capable of generalizing to ITN images and tended to assign cases to the malignant category. This could also explain the discrepancies observed in the ITS 100 system. The commercial ITS 100 system, which examined 1,007 TN US images, exhibited a sensitivity of 92.21%, specificity of 83.20%, and accuracy of 89.97% (30). However, in our ITN cohort, the sensitivity was 70.7%, specificity was 61.4%, and accuracy was 65%. Similarly, the S-Detect unit, an AI model for TNs (31), achieved an accuracy of 81.7% for 454 TNs but only an AUC of 0.795 for 159 ITNs (32). In the current cohort, the Radunion model misclassified 47 ITNs, including 11 benign ITNs and 36 malignant ITNs for pathology. The nodule sizes were evenly distributed from 0.3 to 3.7 cm, 26 Bethesda III nodules with a size distribution of 0.3-3.7 cm, and 21 Bethesda IV nodules with a size distribution of 0.4-2.1 cm.

These AI models were designed to reduce clinical workload and improve the efficiency of junior radiologists (33). One of the primary objectives of US radiomic studies is to avoid unnecessary biopsies in patients with benign nodules. Park et al. (22) combined radiomics with the ACR or American Thyroid Association guidelines (3, 28) and found that all readers showed improved performance and reduced unnecessary fine-needle aspiration (FNA) rates. Huang et al. (27) developed a radiomics nomogram that achieved an unnecessary FNA rate of 18.66% while maintaining an accuracy of 82.48% for TNs. The Thynet-assisted strategy, a well-established method, reduced the number of FNAs from 61.9% to 35.2% in a simulated scenario (23). In this study, we provide evidence that the Radsize model can reduce the unnecessary biopsy rate by up to 48.9% compared to junior radiologists, achieving an unnecessary biopsy rate of 21.1%. These results indicate that US radiomics models hold significant promise in the preoperative diagnosis of ITNs, especially for less experienced radiologists.

This study had some limitations. First, the proportion of malignant nodules in the entire cohort of ITNs from a single medical center (71.1%, 140/197) was higher than that reported in other studies (19, 22), potentially introducing selection bias, such as lower specificity and lower NPV. The proportion of malignant cases would influence the generalizability and robustness of models among other dataset. Diagnostic thresholds for models may be low, leading to reduced predictive power for low-risk populations. Models may be overfitted to high-risk characteristics, making it difficult to accurately identify people at average risk. No more populations for external validation is the second shortage. The majority of patients at our center present with higher-risk nodules and tend to prefer ablative therapy when the nodular size <10 mm (34), some patients with nodular size >10 mm also require to try ablation therapy, leaving the patients with bigger or more risky nodule have to undergo the surgery, which results in a higher percentage of malignant nodules among those undergoing surgery. To address this limitation, collaboration across multiple medical centers is needed to further optimize and validate the performance of these radiomics models by different populations. Meanwhile, the retrospective design and potential variability in US image acquisition also effects the results. Thus, collecting row data in prospective research and expanding the range of imaging data, including contrast-enhanced US, microvascular imaging, and super-resolution US, is necessary. Combining multiple-modality models will be promising in improving diagnostic performance and minimizing unnecessary biopsies for ITNs (35, 36). Finally, we should consider further optimization and applicability studies for the model performance. We should establish clear conditions for the applicability of such a model in the clinical process and the management of its use, including a systematic training program for its users.

## 5 Conclusions

The US radiomics models developed in this study, particularly the Radsize and Radunion models, demonstrate the potential to serve as convenient and accurate adjunct tools for predicting malignancy in ITN. These models can significantly enhance diagnostic performance, particularly for junior radiologists, by improving accuracy and reducing unnecessary interventions, such as biopsies and surgeries. Our findings highlight the broader implications of adopting radiomics-based approaches in clinical practice, including more standardized diagnoses and improved patient management. Future studies should prioritize validating these models across diverse populations and integrating additional imaging modalities, such as contrast-enhanced and super-resolution US, to further optimize their diagnostic capabilities.

## Data Availability

The original contributions presented in the study are included in the article/**Supplementary Material**. Further inquiries can be directed to the corresponding authors.
