# Supplementary figures and images for: Ultrasound radiomics models improve preoperative diagnosis and reduce unnecessary biopsies in indeterminate thyroid nodules
Source: Front Endocrinol (Lausanne). 2025 Jul 10;16:1615304. doi: 10.3389/fendo.2025.1615304 (PMC12286800; doi:10.3389/fendo.2025.1615304)

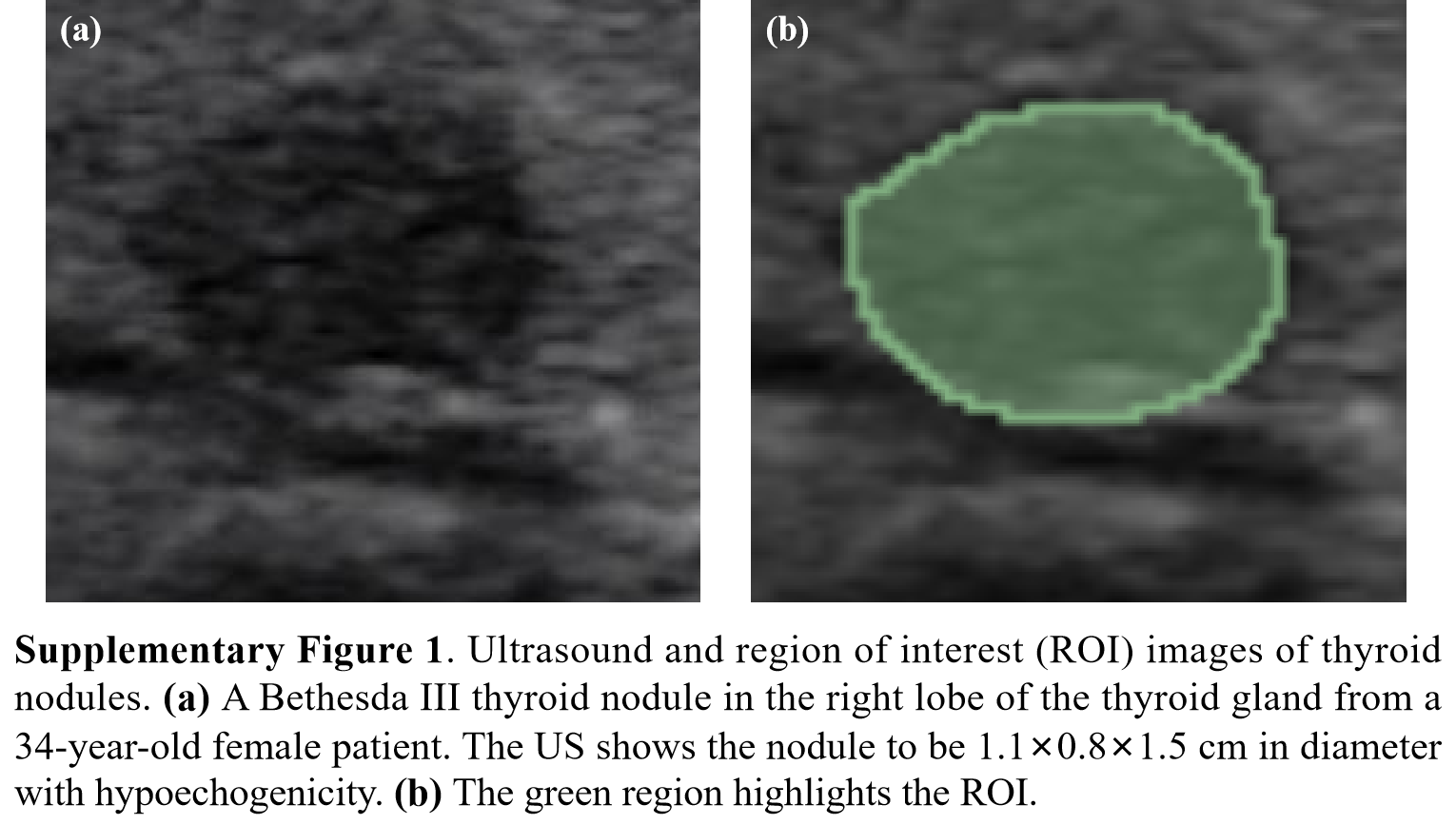

Supplement: Supplementary file 1 [file Image1.tif]

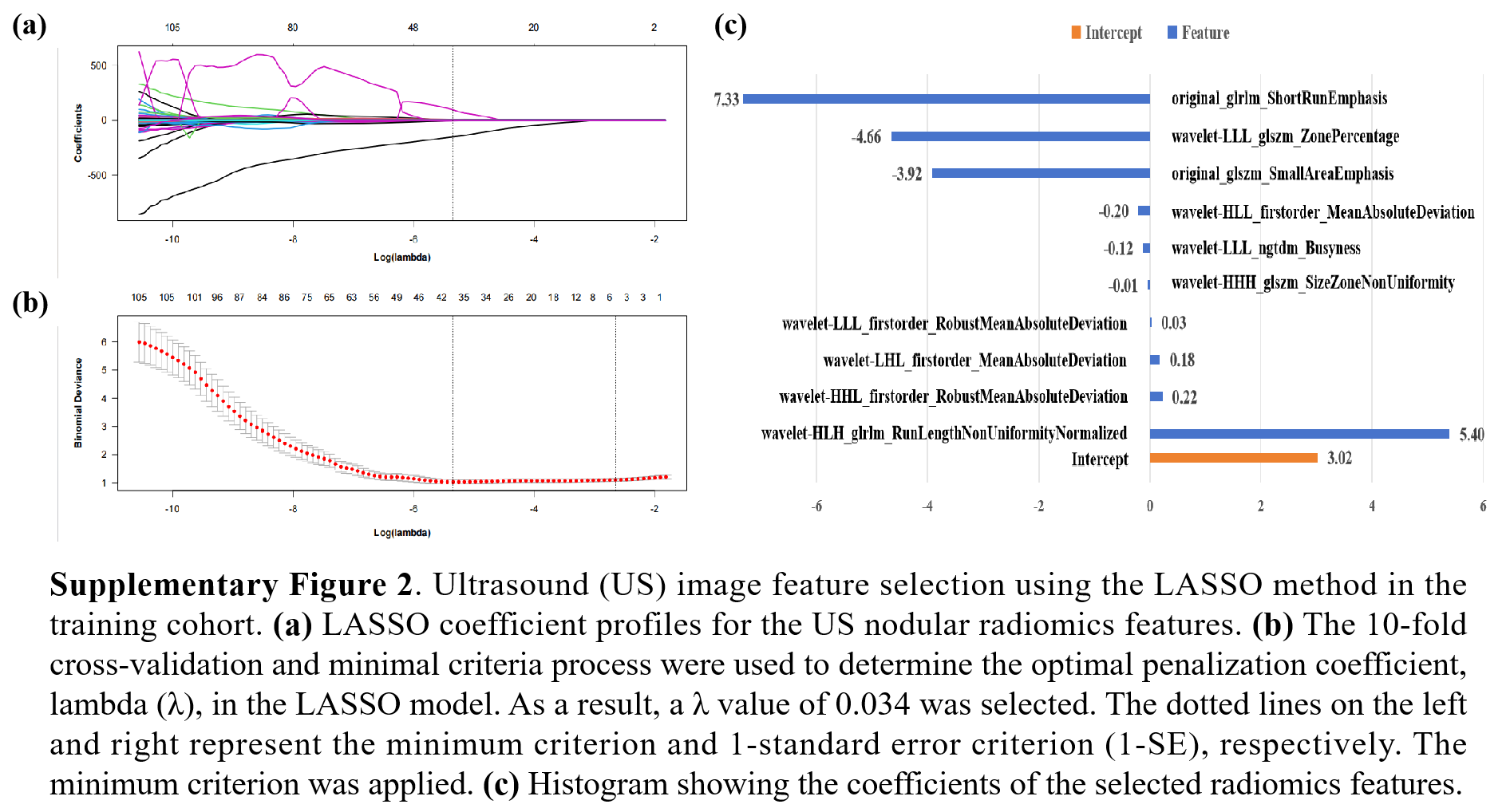

Supplement: Supplementary file 2 [file Image2.tif]
